# What constitutes effective problematic substance use treatment from the perspective of people who are homeless? A systematic review and meta-ethnography

**DOI:** 10.1186/s12954-020-0356-9

**Published:** 2020-01-31

**Authors:** Hannah Carver, Nicola Ring, Joanna Miler, Tessa Parkes

**Affiliations:** 1 Salvation Army Centre for Addiction Services and Research, 4T26, RG Bomont Building, Faculty of Social Sciences, University of Stirling, Stirling, FK9 4LA UK; 2 School of Health and Social Care, Sighthill Campus, Edinburgh Napier University, Edinburgh, EH11 4BN UK

**Keywords:** Substance use, Treatment, Interventions, Qualitative, Homelessness, Meta-ethnography, Inclusion health

## Abstract

**Background:**

People experiencing homelessness have higher rates of problematic substance use but difficulty engaging with treatment services. There is limited evidence regarding how problematic substance use treatment should be delivered for these individuals. Previous qualitative research has explored perceptions of effective treatment by people who are homeless, but these individual studies need to be synthesised to generate further practice-relevant insights from the perspective of this group.

**Methods:**

Meta-ethnography was conducted to synthesise research reporting views on substance use treatment by people experiencing homelessness. Studies were identified through systematic searching of electronic databases (CINAHL; Criminal Justice Abstracts; Health Source; MEDLINE; PsycINFO; SocINDEX; Scopus; and Web of Science) and websites and were quality appraised. Original participant quotes and author interpretations were extracted and coded thematically. Concepts identified were compared to determine similarities and differences between studies. Findings were translated (reciprocally and refutationally) across studies, enabling development of an original over-arching line-of-argument and conceptual model.

**Results:**

Twenty-three papers published since 2002 in three countries, involving 462 participants, were synthesised. Findings broadly related, through personal descriptions of, and views on, the particular intervention components considered effective to people experiencing homelessness. Participants of all types of interventions had a preference for harm reduction-oriented services. Participants considered treatment effective when it provided a facilitative service environment; compassionate and non-judgemental support; time; choices; and opportunities to (re)learn how to live. Interventions that were of longer duration and offered stability to service users were valued, especially by women. From the line-of-argument synthesis, a new model was developed highlighting critical components of effective substance use treatment from the service user’s perspective, including a service context of good relationships, with person-centred care and an understanding of the complexity of people’s lives.

**Conclusion:**

This is the first meta-ethnography to examine the components of effective problematic substance use treatment from the perspective of those experiencing homelessness. Critical components of effective problematic substance use treatment are highlighted. The *way* in which services and treatment are delivered is more important than the *type* of treatment provided. Substance use interventions should address these components, including prioritising good relationships between staff and those using services, person-centred approaches, and a genuine understanding of individuals’ complex lives.

## Background

The number of people experiencing homelessness is significant and growing, with estimates of 307,000 people in the UK [1], 550,000 in the USA [2] and 235,000 in Canada [3] at any one point. Homelessness is a complex and multi-faceted issue. Individuals who are homeless are those without suitable, stable and permanent housing, including those who are sleeping rough, living in hostels, with family/friends or in residential treatment programmes; living in insecure accommodation; and those living in unsuitable housing [4]. Homelessness is caused by a range of factors, with poverty often being central in the likelihood of someone becoming homeless [5, 6]. The experience of other adverse events, such as traumatic childhood experiences, imprisonment, institutional care, substance use, relationship breakdowns and mental health problems are also associated with increased likelihood of homelessness [7–9]. Homelessness, housing and health are intrinsically linked [10]. People experiencing homelessness report poorer physical and mental health than the general population [11]. For instance, in the UK, this group are four times more likely to die prematurely; on average, aged 37 years for women, and 42 years for men [12].

There are strong links between homelessness and substance use [13], with a significant number of people experiencing homelessness using alcohol and/or drugs [14]. For some, it is a way of coping with previous trauma, by blocking out disturbing memories and emotions; for some, a habit they may have acquired prior to becoming homeless; and for others, still a new experience they may have come across since losing their home [13–16]. Previous research has also demonstrated associations between drug overdose and poverty [17]; housing [18]; and living in single room occupancy, low-income hotels [19]. In the UK in particular, the interconnected issues of homelessness, substance use, mental health problems, violence, trauma and criminal justice involvement are particularly strong [6, 20]. While there are strong links between homelessness and substance use, especially among street-involved populations, it is important to note that not all people experiencing homelessness will use drugs and/or alcohol. The focus of this review is, however, on these individuals who are experiencing both homelessness and problematic substance use.

Despite increased morbidity and mortality, engagement with healthcare often occurs at crisis point, where those experiencing homelessness use accident and emergency services rather than primary care [21], with high cost implications [22, 23]. Avoidance of mental health services and problems taking medication as required are also reported by those experiencing homelessness, often due to a range of external barriers [24]. Recent austerity measures and funding cuts to services have resulted in reduced services, and services discharging people more quickly [6, 25–27], making access to health care and problematic substance use treatment more challenging. The importance of taking a holistic approach to supporting those who are homeless with problematic substance use is acknowledged. For example, several quantitative systematic reviews have examined the effectiveness of different types of interventions for this group, and these conclude that provision of housing [28], tailored primary care services [29] and formal case management [28, 30, 31] are effective in improving health/addressing problematic substance use.

### Treatment for problematic substance use

Treatment approaches for problematic substance use treatment are wide ranging and can be placed on a continuum ranging from harm reduction to abstinence-based approaches, with increasing acknowledgement of the value of taking a combined approach [32]. Abstinence-based approaches, where people are expected to completely stop using substances, can be unrealistic for those experiencing homelessness [33–35]. Harm reduction approaches aim to minimise harms/risks associated with problematic substance use [36] and such models are currently recommended for people who are homeless and unable (or unwilling) to work towards abstinence [34, 37–39]. The principles of harm reduction include providing compassionate care that promotes dignity, whilst taking a participative, pragmatic, goal-setting approach [36, 40].

There is limited evidence regarding how treatment for problematic substance use is best delivered to those experiencing homelessness, although engaging, flexible services are important [34, 41]. However, many people with experience of homelessness report that their needs are not well met when accessing mainstream health or substance use services. Stigmatising and negative attitudes also, unfortunately, remain commonplace [21, 42–44]. For those who have successfully engaged with treatment, there can be distinct challenges associated with continued engagement with treatment and recovery as a result of being homeless. Recovery capital refers to the resources people can draw on to begin and maintain recovery from problematic substance use [45] and involves four key components: social capital (relationships with others); physical capital (income, savings, property); human capital (knowledge, skills, health, education); and cultural capital (values, beliefs and attitudes that promote social norms) [45]. Those experiencing homelessness often have low levels of recovery capital [46], which means that the process of treatment and recovery can be particularly difficult, with improvements hard to sustain.

### Qualitative evidence synthesis

Most of the evidence identifying components of effective treatment of problematic substance use comes from quantitative studies examining effectiveness of treatments in terms of quantifiable, post-treatment outcomes. Qualitative research can supplement quantitative research by providing in-depth understanding of service contexts [47], and enabling the needs, preferences and experiences of those using services to be taken more fully into account when developing and evaluating new interventions/services [48]. While individual qualitative studies can provide important personal insights into effective substance use treatment, this evidence is frequently criticised as sitting low in the evidence ‘hierarchy’ [49], and thus its potential contribution to assessing intervention effectiveness is devalued. Synthesising qualitative studies can strengthen the weight of such evidence by bringing together single studies, and there has been recent recognition of the value of such work [50]. Previous qualitative syntheses in the fields of homelessness and problematic substance use have examined the perspectives of mothers in caring for children in shelters [51], their views on treatment services [52, 53] and natural recovery from alcohol and drug problems [54], views of mothers on and safer environments for injecting drug users [55]. This is the first published qualitative synthesis to examine what constitutes effective treatment for problematic substance use from the perspective of people who are homeless.

## Methods

### Meta-ethnography rationale

Noblit and Hare’s [56] meta-ethnography (ME) is the most commonly cited qualitative synthesis approach [57–59]. ME has the potential to produce novel conceptual understandings of complex issues [60] through translating and synthesising original participants’ views/experiences, and authors’ interpretations (as reported in published studies), into a new higher level interpretation, leading to the development of a new theory, model or framework [56]. As such, ME was the most suitable approach for this review. There are seven overlapping phases of ME: (1) getting started; (2) deciding what is relevant; (3) reading the studies; (4) determining how the studies are related; (5) translating studies into one another; (6) synthesising translations; and (7) expressing the synthesis [56]. Although initially devised to include only ethnographic studies, ME has evolved to include all types of qualitative research and is widely used in health research [52]. The methods are outlined below and have benefitted from the newly available eMERGe ME reporting guidance [61]. Further details are presented in Additional file 1.

In phase 1, we identified our ME research question as: *What components of problematic substance use treatment are perceived to be effective by adults* (*aged 18*+) *who are homeless*? Preliminary searching was conducted to ensure the availability of a body of literature to be synthesised. As there are no standard definitions of homelessness [62], we defined our key terms. ‘Homelessness’ was defined as a lack of suitable, stable and permanent housing, and ‘at risk of homeless’ included those likely to lose their homes. Both harm reduction and abstinence-based approaches were considered ‘treatment’; and ‘effectiveness’ was broadly used to mean whatever the recipients of a service or intervention considered was beneficial or helpful to them (see also Additional file 1 for details). The study protocol was developed and registered with PROSPERO (CRD42017069745).

### Search strategies

Systematic literature searching of electronic databases and ‘grey’ literature was conducted in May 2019 (phase 2) to identify relevant studies consisting of comprehensive electronic database searches and ‘grey’ literature searches. The SPIDER tool [63] was used to identify search terms (Table 1 and Additional file 1). Eight electronic databases (CINAHL; Criminal Justice Abstracts; Health Source; MEDLINE; PsycINFO; SocINDEX; Scopus; and Web of Science) were searched for qualitative studies published between 2000 and 2019. ‘Grey’ literature was identified by searching the websites of various relevant organisations for possible items such as research reports published since 2007 (Table 2). Reference lists of all included studies were also reviewed for potential items.

**Table 1:** Search terms identified using the SPIDER tool [55]

**S**ample (service users)	homeless* OR underhouse* OR roofless* OR street involved OR rough sleeping OR unstabl* hous* OR housing instability OR precarious* hous*
**P**henomenon of **I**nterest (perceptions of effective treatment for problem alcohol and/or drug use)	Substance *use OR drug *use OR alcohol *use OR problem* substance use OR problem* alcohol use OR problem* drug use OR addiction OR substance dependenc* OR alcohol dependenc* OR drug taking OR drug dependenc* treat* OR intervention OR recovery OR therap* service*
**D**esign/**E**valuation/**R**esearch type (qualitative)	Qualitative OR focus group OR interview* OR ethnograph* OR observation*

**Table 2:** Organisations included in search for grey literature

Scotland	UK	International
Alcohol Focus Scotland https://www.alcohol-focus-scotland.org.uk/	The Salvation Army https://www.salvationarmy.org.uk/	National Drug and Alcohol Research Centre, Australia https://ndarc.med.unsw.edu.au/
NHS Health Scotland http://www.healthscotland.scot/	Alcohol Change UK https://alcoholchange.org.uk/	National Institute on Drug Abuse, USA https://www.drugabuse.gov/
Alcohol and Drug Partnerships https://www2.gov.scot/Topics/Health/Services /Alcohol/treatment/ADPcontactlist	Society for the Study of Addiction https://www.addiction-ssa.org/	National Institute on Alcohol Abuse and Alcoholism, USA https://www.niaaa.nih.gov/
Institute for Research and Innovation in Social Services https://www.iriss.org.uk/	Public Health England https://www.gov.uk/government/organisations/ public-health-england	Canadian Institute for Substance Use Research, Canada https://www.uvic.ca/research/centres/cisur/
Scottish Drugs Forum http://www.sdf.org.uk/	Pathway/Faculty of Homeless and Inclusion Health https://www.pathway.org.uk/	Centre for Social Research in Health, Australia https://www.arts.unsw.edu.au/csrh
Scottish Government https://www.gov.scot/	Addaction https://www.addaction.org.uk/	Homeless Hub, Canada https://www.homelesshub.ca/
Scottish Health Action on Alcohol Problems https://www.shaap.org.uk/	Crisis https://www.crisis.org.uk/	European Observatory on Homelessness https://www.feantsaresearch.org/
NHS Healthcare Improvement Scotland http://www.healthcareimprovementscotland.org/	Shelter https://www.shelter.org.uk/	
University of Stirling Online Addictions Library https://www.onlinelibraryaddictions.stir.ac.uk/	Royal College of Psychiatrists https://www.rcpsych.ac.uk/	
	Royal College of Physicians https://www.rcplondon.ac.uk/	
	British Psychological Society https://www.bps.org.uk/	
	Groundswell https://groundswell.org.uk/	
	St Mungo’s https://www.mungos.org/	
	Homeless Link https://www.homeless.org.uk/	

### Selection criteria and quality appraisal

Papers were eligible for inclusion if they: (a) reported primary qualitative research of perspectives of treatment for problematic substance use; (b) were published in English; and (c) included adults aged 18 or over who were homeless/at risk of homelessness and had accessed treatment for problematic drug and/or alcohol use. Papers that specifically focused on ‘youth’ homelessness were excluded because they were not comparable to the rest of the literature, in terms of service settings and demographic characteristics of participants. Full inclusion and exclusion criteria are provided in Table 3. Possible items for synthesis were screened by two reviewers (HC/JM) working independently and then comparing results. Items were initially screened against our study eligibility criteria by title and abstract and then full text. Potential disagreements about studies for inclusion were referred for arbitration (TP/NR). Literature searching and screening results were reported using PRISMA [64].

**Table 3:** Study inclusion and exclusion criteria

Inclusion criteria	Exclusion criteria
Adults (aged 18+) who were homeless (or at risk of homelessness) and had accessed treatment for problematic drug and/or alcohol use (currently or in the 10 years prior to the study being conducted).	Participants other than adults (aged 18+) who were homeless (or at risk of homelessness) who had accessed treatment for problematic drug and/or alcohol use more than 10 years ago.
Published studies reporting primary qualitative research studies (any type) with sufficient rich data for synthesis.	Studies not reporting primary qualitative research studies (e.g. surveys, qualitative evidence syntheses). Studies using qualitative methods but which did not report sufficiently rich data for synthesis, e.g. mixed methods research where qualitative data were not presented separately.
Studies published from 2000 in English language.	Qualitative research reported out with these years and not in English language.
Studies that reported participants’ views/experiences of receiving treatment for problematic substance (drugs and alcohol of any type) use only.	Studies that did not report participants’ views/experiences of receiving treatment for problematic substance use. Studies that focused on substances other than drugs and alcohol (e.g. tobacco) or other types of addictions. Studies that included participants with dual diagnoses (e.g. problematic substance use and mental health problems). Studies that only reported the views of others (e.g. service providers).

Studies meeting the inclusion criteria were read repeatedly (phase 3) and quality assessed using the Critical Appraisal Skills Programme (CASP) checklist [65] (see Additional file 2 for the quality appraisal). Quality appraisal allowed for the systematic consideration of study strengths and weaknesses [50]: it was not used to exclude studies [66].

### Data extraction and analysis

**Fig. 1:**
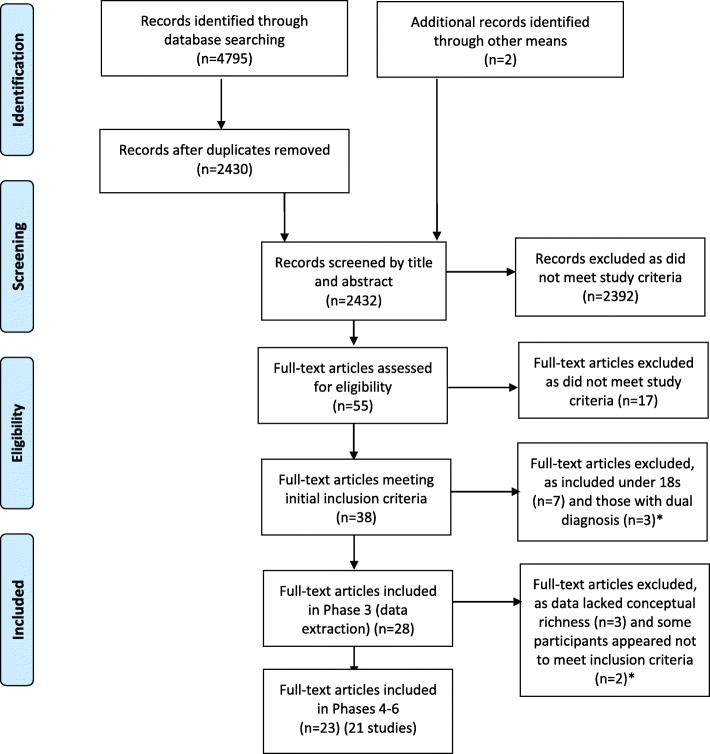
PRISMA diagram. * see Additional file 2: Table S2 for details

Study characteristics, including setting, participant characteristics and methods, were entered into an Excel spreadsheet. First-order (participant quotes) and second-order (author interpretations) data were extracted and entered into NVivo version 11 for each study. As phase 3 progressed, it became apparent that some included studies lacked the depth of reporting of original participant views needed for a ME approach and, as such, would not be suitable for synthesis. A team meeting was held to review the included papers and make a decision for each paper as to whether it had sufficiently rich data for synthesis. Papers which were considered to insufficiently report rich data either had too few participant quotes (five or less) or had more quotes but these were too briefly reported. These papers were therefore excluded from further phases of the ME (see Fig. 1 and Additional file 3).

In phase 4, papers with sufficiently rich data had their first- and second-order data coded line-by-line to identify each study’s main themes and concepts. Resulting codes for first- and second-order data, such as ‘support’, were entered into Excel matrices, which enabled us to determine how studies related (or not) in their design (e.g. participants, or setting) and findings. This facilitated translation (phase 5), as the matrices determined whether similar concepts, themes and metaphors were reported in different studies, albeit expressed in different language (reciprocal analysis), and enabled identification of disconfirming cases (refutational analysis), i.e. studies that reported findings different from others.

Study findings were also reciprocally translated against two a priori categories created from the review question: (1) ‘*what* components of treatments/interventions were perceived by study participants as effective, and why?’; and (2) ‘*how* does effective treatment work?’. Through an iterative process of translation, concept maps (Additional file 4) were created from which an ‘over-arching’ third-order interpretation was developed and formed into a new line-of-argument (phase 6) and conceptual model. In phases 3–5, HC led on data extraction and analysis, with NR/TP/JM checking for accuracy. Any disagreements were discussed until consensus was reached, with regular team meetings used for reflection, critical in ME, allowing us to challenge analytical processes and interpretations. In phase 7 (expressing the synthesis), the initial narratives of our line-of-argument and conceptual model were presented for ‘sense checking’ to three people with lived experience of homelessness and substance use. Their comments were carefully reflected upon in the context of the study data and, where appropriate, refinements were made to our synthesis and conceptual model. We also reflected on those papers which were excluded from translation because of insufficiently rich data (*n* = 3). This was done to consider whether they would have altered or refuted our final interpretation had we included these studies in phases 4–6.

## Findings

### Overview of included studies

Our searches identified 23 papers (Fig. 1): 22 published papers [33, 67–87], and one ‘grey’ literature study [88]. Four papers were from two studies [68, 69, 77, 85], meaning the findings from 21 studies were synthesised.

Characteristics of the included papers are detailed in Table 4. In this table, we highlight the differences in these included studies, in terms of setting, participant demographics and intervention/service. Briefly, the 21 studies were conducted between 2002 and 2018 in the USA (*n* = 11), Canada (*n* = 7) and the UK (*n* = 3), involving 462 participants (one study did not report participant numbers) ranging from 18 to 62 years. Three studies focused entirely on women [84, 86, 87], and five involved men only [70, 80–83]. In the remaining studies, although women were involved, 50% to 84% of participants were male; only one study reported a majority of female participants [75].

Participants’ ethnicity was reported in 17 studies, and, in 11 of these, most participants were from ethnic minority groups. For studies conducted in the USA, most participants were Black, Hispanic, mixed race or Indigenous American. In four of the five Canadian papers that reported ethnicity, most participants were Indigenous Canadian. In one UK study, participants were Polish [88]. Data were collected using individual interviews (*n* = 13), focus groups (*n* = 2) and combined methods, including interviews, focus groups and town hall meetings (*n* = 6). Participants were recruited from a range of services rather than directly from the streets. Ten studies provided insight into participant views of services generally [33, 67, 72, 75, 76, 82, 84, 86–88], one study explored a hypothetical intervention [74] and tenstudies examined specific substance use interventions [68–71, 73, 77–81, 83, 85].

**Table 4:** Characteristics of included studies (chronological order)

Authors	Country	Substance	Setting	Participant information	Methods	Key findings
Neale and Kennedy (2002) [67]	UK	Drugs	Hostels/drug agencies	*N* = 36; average age 25 years; 50% female; none in employment; many spent time in institutions; most marginally housed.	Individual semi-structured interviews to explore experiences of and barriers to accessing services. Analysis: Framework method.	Range of factors viewed as good practice in terms of services, with emphasis on staff attitudes and services offered.
Lee and Petersen (2009) [78]	USA	Alcohol and drugs	Drop in centre	*N* = 15; average age 43 years; 60% male; 60% Black; all homeless.	Individual semi-structured interviews to explore experiences of treatment and marginalisation. Analysis: Grounded theory	Positive outcomes in terms of demarginalisation; engagement; quality of life; social functioning; change in substance use; and articulation of future goals/plans.
Rayburn and Wright (2009) [80]	USA	Alcohol	Men’s shelter	*N* = 10; aged 40s–50s; all men experiencing homelessness/problem alcohol use; 80% Black; 50% completed high school	Life history interviews to explore men’s moves from active addiction to recovery and process of becoming AA member. Analysis: Variant of grounded theory	Participants experienced four types of barriers to sobriety/being part of AA when experiencing homelessness. These barriers were identification with AA; sponsorship; step work; and time constraints.
Rayburn and Wright (2010) [81]	USA	Alcohol	Men’s shelter	N =?; all men experiencing homelessness/problem alcohol use	Individual unstructured interviews exploring recovery and experience with AA. Analysis: No detail	Study uncovered some ways homeless men achieve and maintain sobriety; adapting concepts of 12 step programmes to homeless men, shows need for flexible approach.
Burkey et al. (2011) [82]	USA	Alcohol and drugs	Residential therapeutic community for men	*N* = 10; all men; average age 43 years; all Black; all homeless.	Individual semi-structured interviews to explore social ties in recovery from substance use. Analysis: Miles and Huberman approach	Identified three types of social ties: family, recovery network and outside relationships: importance of relationships with peers, 12 step sponsors and counsellors, recovery network key; also relationships with healthcare professionals
Kidd et al. (2011) [83]	Canada	Alcohol	Managed Alcohol Program	*N* = 1; male; aged 48 years, experiencing homelessness and had many failed attempts at abstinence.	Individual semi-structured interviews at 3 time points with one man to develop case study of experiences. Analysis: Grounded theory/narrative coding	Positive experience of MAP, strengths of staff (caring), benefits of alcohol administration, peaceful environment. Feeling at home, knowing residents.
Sznajder-Murray and Slesnick (2011) [84]	USA	Alcohol and drugs	Emergency shelter for families	*N* = 28; all women; average age 29 years; 61% Black; all had children (8 had children removed from custody, 3 currently pregnant); all residing in homeless shelter.	Focus groups (×3) to explore needs and experiences of services. Analysis: open and axial coding.	The women talked about how they had been treated differently to how they would like to be treated; highlighted particular issues for women/mothers who are homeless and using substances, particularly in terms of fear.
Collins et al. (2012a) [85]	USA	Alcohol	Project based Housing First	*N* = 17; average age 48 years; 40% white, 27% American Indian; many had experiences of treatment; all living in Housing First program.	Individual interviews and observations to explore views of programme. Analysis: Constant comparative method.	Harm reduction approach of the programme as a key factor in their attainment and maintenance of housing. Most did not see abstinence-based treatment as viable option. Harm reduction approach resulted in their successful reduction in drinking or abstinence in a way that abstinence-based treatments had not.
Collins et al. (2012b) [77]	USA	Alcohol	Project based Housing First	*N* = 17; average age 48 years; 40% white, 27% American Indian; many had experiences of treatment; all living in Housing First program.	Individual interviews and observations to explore views of programme. Analysis: Constant comparative method.	Study highlighted strengths and weaknesses of programme, including transitions into the programme, managing day-to-day life and community building.
Thickett and Bayley (2013) [88]	UK	Alcohol	Alcohol service provider	*N* = 12; all Polish street drinkers; 58% male; aged 33–62 years; all homeless/at risk of homelessness.	Individual semi-structured interviews to explore experiences with services. Analysis: Braun and Clarke’s thematic analysis.	Participants talked about positive and negative experience of treatment including social networks; social services; health services; homelessness services; specialist alcohol service provider; and barriers to service use.
Salem et al. (2013) [86]	USA	Alcohol and drugs	Residential treatment facility	*N* = 14; all women; recently released from prison; average age 42 years; 79% Black; 79% had children; all homeless, living in residential treatment facility.	Focus groups (×2) exploring experiences of challenges experienced in accessing treatment. Analysis: Grounded theory.	Women talked about difficulties in accessing healthcare and other services; lack of support staff onsite; lack of education and criminal record made it difficult to get a job. Strategies to remain sober included feeling empowered, having a job, going to NA/AA meetings, having housing, job skills/education, aftercare program and support.
Baird et al. (2014) [87]	USA	Alcohol and drugs	Outpatient programme for women	*N* = 10; all women; all homeless, living in shelter.	Individual structured interviews to explore ways to maintain abstinence Analysis: No detail.	Four main concerns identified by respondents: lack of communication between service providers; inconsistency in personnel during recovery; inconsistency in relapse policies; clients feeling ill prepared to live in the ‘real world’ after completion.
Neale and Stevenson (2014a) [69]	UK	Alcohol and drugs	Hostels	*N* = 30; average age 43 years; 83% male; 60% white; poly drug use common; most receiving some treatment; all homeless, living in hostels.	Individual semi-structured interviews at 2 time points to explore experiences with computer assisted therapy intervention. Analysis: Framework method.	Computer assisted therapy intervention for drug users in hostels viewed as beneficial in helping with substance use as well as wellbeing and improving skills/confidence. Negative issues were around structural barriers such as location of computers, quality and quantity of equipment.
Neale and Stevenson (2014b) [68]	UK	Alcohol and drugs	Hostels	*N* = 30; average age 43 years; 83% male; 60% white; poly drug use common; most receiving some treatment; all homeless, living in hostels.	Individual semi-structured interviews at 2 time points to explore experiences with computer assisted therapy intervention Analysis: Framework method.	Viewed programme positively, but mentor support was crucial. Need for good relationships with staff to help engage in programme. Also encouraged to have more open/honest conversations. Need for flexible approach. Use within context of therapeutic relationship crucial.
Evans et al. (2015) [70]	Canada	Alcohol	Managed Alcohol Program	*N* = 10; all men; average age 51 years; all had many failed attempts at abstinence; all homeless, living in Managed Alcohol Program; within 1.5 years of study ending, 3 had died.	Individual interviews and follow up focus group (×1) to explore experiences of program. Analysis: No detail	Participants talked about importance of social belonging within programme, mutual support and relationships with support workers as important. Programme allowed increased awareness of alcohol and health and opportunity for self-management.
Clifasefi et al. (2016) [71]	USA	Alcohol	Housing First program	*N* = 44; 82% male; average age 53 years; 43% white; all had severe alcohol problems; all living in single site Housing First program.	Individual semi-structured interviews and observations to explore experiences of program Analysis: Constant comparative method	Participants reported issues with consistency in activities and services; expressed a desire for groups where they could learn about harm reduction; did not want focus to be on abstinence. Participants discussed an aversion to abstinence-based treatments with multiple failed attempts. Many indicated that abstinence was only achieved after entering service with harm reduction focus.
Collins et al. (2016) [33]	USA	Alcohol	Housing agencies	*N* = 50; 84% male; average age 53 years; 46% white; all currently/formerly homeless.	Individual semi-structured interviews to explore experiences of treatment and services. Analysis: Content analysis.	Participants talked about experience of formalised, abstinence based approaches in terms of positives and negatives. Also experience of alternative, self-defined pathways that included basic needs; harm reduction counselling; meaningful activities; social networks; natural recovery.
McNeil et al. (2016) [74]	Canada	Drugs	Hospitals	*N* = 30; 53% male; average age 45 years; 57% Indigenous; most had multiple hospitalisations due to drug use; all ‘structurally vulnerable’/at risk of homelessness.	Individual semi-structured interviews to explore perspectives of hospital based harm reduction. Analysis: Inductive and deductive approach.	Harm reduction approach in hospital settings would allow patients to complete their treatment for health problems and not have to be discharged early because of continued drug use; also mean safer use/risk reduction; harm reduction viewed as reducing stigma, being non-judgemental and having staff who understand/care.
Pauly et al. (2016) [73]	Canada	Alcohol	Managed Alcohol Program	*N* = 7; 57% male; average age 42 years; all Indigenous; had all been in MAP for at least 1 year; experience of chronic homelessness, alcohol use and police contact.	Individual semi-structured interviews to explore experiences of programme. Analysis: Constant comparative approach.	MAP viewed as a place of safety, characterised by caring, respect, trust and non-judgemental attitude, with sense of home and opportunities to reconnect with family.
Perreault et al. (2016) [72]	Canada	Drugs	Peer-run day centre and housing units	*N* = 13; 60% male; aged 30–60 years; half had Hepatitis C/mental health problem; all homeless, living in housing units.	Individual semi-structured interviews and focus group (×1) to explore experiences of programme. Analysis: Thematic analysis	Participants identified several issues in terms of satisfaction and dissatisfaction; length of time (3 years) too short and need for support in returning to education/work. Differences in opinion re. use of peers vs. professional staff.
Chatterjee et al. (2018) [75]	USA	Drugs	Family shelters	*N* = 14; 79% female; average age 35 years; all part of families experiencing homelessness; 64% white; all had diagnosis of opioid use disorder; 86% in treatment.	Individual interviews to explore experience of opioid use disorder and treatment when experiencing homelessness as a family. Analysis: Immersion- crystallisation method	Study highlighted experiences of treatment, barriers and ideal treatment for those experiencing opioid use and homelessness as part of a family.
Crabtree et al. (2018) [76]	Canada	Alcohol	Communities	*N* = 85; no formal details collected but majority men; mostly white or Indigenous; aged 20–50 years; all homeless/at risk of homelessness.	Weekly town hall meetings (×14), steering committee meetings (×7) and follow up focus groups (×4) to explore harm and harm reduction strategies among people who drink non-beverage alcohol. Analysis: Interpretative description.	Participants identified harms and harm reduction strategies they employ, including sharing alcohol, pooling money to buy alcohol, diluting alcohol, drinking alone or with others and looking after one another. Proposed four harm reduction strategies - safe spaces, MAPs, peer based programs and educational programs.
Pauly et al. (2018) [79]	Canada	Alcohol and drugs	Transitional housing programmes	*N* = 16; aged 32–52 years; 56% male; 81% white.	Semi-structured individual interviews conducted to explore implementation of harm reduction in a transitional programme setting. Analysis: Thematic analysis.	Study highlights challenges of settings with harm reduction and zero tolerance approaches to substance use. Harm reduction supplies were available but all substance use was prohibited on site. Despite zero tolerance approach, staff would turn blind eye to use onsite.

Study findings reciprocally translated into our a priori categories as follows.

#### What treatments/interventions are perceived as effective by those using them, and why?

Table 5 provides details of participant experiences with harm reduction and abstinence-based interventions, delivered in different settings. Participants in the study by McNeil et al. [74] discussed the merits of a hypothetical harm reduction intervention drawing on their experiences of other interventions such as Twelve Step programmes.

**Table 5:** Substance use interventions—participant experiences and perceptions of effectiveness

Features reported by participants as being effective or not	Examples of first-order participant data
*Abstinence*-*based programmes*: interventions that required participants to be abstinent from alcohol/drugs, including residential programmes. Twelve Step programmes such as Alcoholics Anonymous or Narcotics Anonymous have a spiritual orientation and advocate complete abstinence although participants take part in various activities including attending meetings and getting a ‘sponsor’. These were discussed in five papers [24, 61, 65, 71, 79].	
(+) Adapting principles to meet needs (+) Desire to help others (+) Peer support (−) Power imbalances (−) Increased urges/ cravings (−) Sense of failure (−) Challenges associated with finding a ‘sponsor’ at AA	‘I wanna be able to help somebody. I wanna be able to start something. If I wanna go to the grocery store, and out of my pocket, buy lunchmeat, cheese, and a couple of cases of soda, go out on a Saturday, where people at, and just hand out food—I wanna be able to do that’ (Participant in Rayburn and Wright [61]) ‘I’ve gone to AA, and it does help because you are around like-minded people’ (Participant in Collins et al. [65]) ‘I went to [Narcotics Anonymous] and this guy was talking about how his pockets were turned inside out looking for crack…I had a using dream of crack after listening to his thing. So…I just really did not want to go back’ (Participant in Clifasefi et al. [71]) ‘Oh, this ‘AA all the way,’ and ‘the only way to stay sober is AA’ … There are other ways to stay sober … And, you know, you just feel like when you go to AA, you feel like you are a failure’ (Participant in Collins et al. [24]) ‘Getting a sponsor. I got one, but I struggle with it. I had a real deep struggle with it, because at first I said, “I’m not getting no sponsor man.” For me to get a sponsor, is just like saying, I do not trust in my higher power. And then a sponsor is just a human being, just like me. You know, I’m not gonna have nobody telling me … you not ready for no relationship …I just wasn’t ready for that’ (Participant in Rayburn and Wright [79]).
*Housing*-*based harm reduction*: Managed Alcohol Programmes provide regular doses of alcohol with supported housing and wider care provision [63, 70, 73] and help users manage/reduce unpleasant and potentially fatal alcohol withdrawals. In Housing First settings participants are provided with accommodation where alcohol use is tolerated [65, 71, 77]. Housing First refers to programmes which provide “low-barrier, non-abstinence-based, immediate, supportive and permanent housing to chronically homeless people who often have co-occurring substance use and/or psychiatric disorders” [65]. Transitional housing programmes provide support in helping people move out of homelessness and those with a harm reduction approach may be more beneficial than those expecting abstinence at entry [78].	
(+) Having a home (+) Managing withdrawal symptoms (+) Safety (+) Peer support (+) Non-judgemental staff (−) Availability of alcohol when wishing to be sober (−) Challenges associated with settling into a new, unknown environment (e.g. MAP/housing programme), such as getting to know peers and staff	‘You know sometimes you do not drink that much but it’s enough to get you well—to stop the shakes’ (Participant in Collins et al. [65]) ‘It has helped me a lot you know; where I used to drink heavy and now I slowed down a lot. Right?’ (Curtis, in Evans et al. [70]) ‘I’m starting to feel very comfortable now. Putting my pictures up. .. makes me feel at home…I can relax a little better because I know the people’ (Mark in Kidd et al. [63]) ‘Like I went out last week and I ended up using... I came back and I talked about it and I have not used all week, which is great. But they are there for me whether I do, whether I do or I do not’ (Participant in Pauly et al. [78]) ‘Yeah, we think of each other as a family. When there’s a new person that comes in we welcome them with arms open. And we see they need to be [guided] for the first couple of weeks and we take them and we teach ‘em. And we, ah, show them around and if they need something I’ll show them where to get it, where to ask for it’ (Participant in Pauly et al. [73]) ‘… it’s hard to stop [drinking]. I mean it’s hard to stop here, you know what I mean? Because … [if] I do not have [alcohol], somebody else does. People invite you to come along and all that other kind of things … and it’s hard” (Participant in Collins et al. [65]) “I do not know anybody who do not have fear, you know? What happens if I lose this place, you know? Am I gonna go back home to [name]? I do not wanna go to treatment. I did nothing bad’ (Participant in Collins et al. [77])
*Harm reduction interventions delivered online*: Breaking Free Online is a tailored intervention for men and women experiencing homelessness and problematic substance use. It provided users with a 12-week computer-assisted psychological treatment alongside ‘real world’ staff mentor support [68, 69]. It offered various strategies aimed at helping people identify, understand and actively address the psychosocial and lifestyle factors underpinning their substance use, without requiring abstinence.	
(+) Flexibility, easily accessible, non-judgemental, user friendly (+) Prompts to have conversations with staff (+) Development of new skills (including computing) and routine (+) Increased awareness of substance use (+) Development of coping strategies (−) Lack of privacy, poor equipment, lack of availability of staff	‘The convenience of it for starters. I mean, it can be done in the hostel, it can be done in my bedroom…it can be done anywhere, if you have got a laptop. You can do it in the middle of the park somewhere on a nice summers day, rather than going all the way to [drug agency], catching the bus and travelling all the way up there’ (Trent, in Neale and Stevenson [69]) ‘I am doing my daily routine quite well, making sure I get up in the morning and do not just stay up watching shit TV until like four o‘clock in the morning. So I think I’m better now, better equipped to get up and do something during the day, like a normal human being’ (Sarah, in Neale and Stevenson [68]) ‘It [BFO] gives me the ability to talk about my emotions, about me, to [name of mentor]…I am just becoming more open, and, as I said, which it helps me to open up to him’ (Leona, in Neale and Stevenson [68]) ‘There is always somebody on them [computers]…I have not really had the head space to get on and concentrate, you know. I would like to, but there is always somebody shouting or screaming or bawling, you know, and I want to get on it, you know, but I just cannot get the space to’ (Thomas, in Neale and Stevenson [68])

(+) = Components of these interventions that participants found to be effective (i.e. beneficial or liked)

(−) = Components of these interventions that participants found to be ineffective (i.e. disadvantageous or disliked)

Abstinence-based treatment was praised for the provision of peer support and people’s desire to help others, with one participant stating: ‘*it does help because you’re around like-minded people*’ ([27]; p. 91). Abstinence-based residential treatment was the ‘*time out*’ from heavy alcohol use and homelessness, with some using it as a safe space to stop drinking for a short period, because ‘*Treatment wasn’t really about getting sober*’ ([27]; p. 91). Some said that they felt better after enforced abstinence [33]. Less positive, however, was the perception that abstinence-based approaches were not effective because they triggered cravings [78, 83, 85], and did not address the underlying issues affecting substance use and homelessness [33, 71, 73, 74, 78]. Some stated that these approaches were ineffective because they were unable/unwilling to stop using substances, or if they abstained during their programme they returned to using substances on leaving [33, 71, 78].

Housing programmes involving harm reduction approaches, such as Housing First, Managed Alcohol Programmes and transitional housing, were viewed as providing a place of safety and security, with people feeling at home: ‘*you feel safe, you feel like you’ve got a warm place to stay, and you know, some home*’ ([73]; p. 8). The provision of alcohol within Managed Alcohol Programmes and allowing people to use alcohol within Housing First settings, ensured that withdrawal symptoms could be controlled [73, 83, 85]. Participants also spoke highly of the peer support element of these settings, being around those who have similar experiences and the importance of non-judgemental staff [33, 73, 85]. On the other hand, the availability of alcohol when someone wanted to stop using alcohol was perceived as challenging [33, 71, 85].

Harm reduction approaches were discussed by participants in six studies [33, 70, 73, 74, 79, 83], although, in one case, the intervention which allowed those who use drugs to do so safely in hospital was hypothetical [74]. Participants appreciated trusting, non-judgemental staff, the peer support available to them and being in a place of safety [73, 74, 79, 83]. Reducing alcohol consumption and managing withdrawals from alcohol were also described positively [73]. Some participants spoke of the challenges of being in an environment where alcohol was available when they were keen to avoid drinking: ‘*If there is a group of people that are drinking a whole bunch … I reach a certain point, and I’ll excuse myself*’ ([33]; p. 94). The tensions between harm reduction and abstinence in a transitional housing setting were discussed, highlighting the mixed messages and confusing policies participants received in a service in which while abstinence was not required, substance use was forbidden on site [79].

In terms of online delivery of harm reduction interventions, the flexibility, user-friendliness and non-judgemental approach of one such intervention, Breaking Free Online, was reported positively by participants [68, 69]. Participants also appreciated the opportunity to develop new skills, such as using computers, and coping strategies:

*The convenience of it … it can be done anywhere, if you have got a laptop. You can do it in the middle of the park somewhere on a nice summer’s day, rather than going all the way to [drug agency], catching the bus and travelling all the way up there*. ([68]; p.260–261)

However, participants also described occasions when this intervention was less effective, including difficulties in using the programme in communal areas, on poor equipment, or when staff were unavailable to allow access [69].

The findings from these qualitative studies cannot conclude that these interventions are effective for all users, at all times, but offer useful insights into the particular elements of these programmes that participants found beneficial. Further details of participant views of what worked (and did not) are shown in Table 5.

#### How does effective treatment work from the perspective of people who are homeless?

Across all studies, five factors were identified regarding *how* substance use treatment was perceived as effective for those experiencing homelessness: facilitative service environments; compassionate and non-judgemental support; the importance of time; having choices; and opportunities to (re)learn how to live.

(i) Facilitative service environments

Participants in 11 studies [67–69, 71–74, 76, 78, 82, 84, 88] discussed the service environment and how it affected their experiences of treatment. In most studies, participants reflected on the positive service environments within harm reduction-oriented services. Service environments could be described as ‘facilitative’ where they had features that enabled health, wellbeing and elements of recovery for those using them. For example, Neale and Kennedy’s [67] participants identified facilitative service environments as those that are friendly, relaxed, clean, warm and offer privacy. Service environments were welcoming when they did not result in (re-)traumatisation by creating feelings of powerlessness [78]. These service environments had staff who were understanding of individual circumstances, well-trained, available and had a respectful attitude:

*It’s almost like they’re giving all their trust in you, the workers here, it’s like they trust you … got confidence in you*. ([73]; p. 6)

Such positive environments included settings where participants had access to staff who had lived experience of homelessness, substance use or other relevant life events, suggesting that they believed that such staff could relate to them more effectively [72, 78, 82, 84].

The importance of safety was also reported by participants in three harm reduction studies [73, 74, 83], as illustrated below:

*People carry knives, there’s fights every night. People are drinking that hairspray and mouthwash … But here that doesn’t happen … it’s a big difference … Yeah, I felt a lot safer.* ([73]; p. 5)

Conversely, male and female participants talked about sub-optimal service environments where they felt unable to trust providers [73, 84], did not have access to staff trained in dealing with challenging behaviours or who understood drug and alcohol use [71, 74] or experienced high staff turnover [67]. In Sznajder-Murray and Slesnick’s [84] study, women reported being fearful of having their children removed, and viewed staff as lacking understanding, and being judgemental, disrespectful and disregarding of their own efforts to manage their problems. In another study, women stated that their relationship with staff in an abstinence-based setting was adversely affected by high staff turnover, with participants finding it difficult to explain their situation again to new staff: ‘you can’t pour it all out again’ ([87]; p. 405).

(ii) Compassionate and non-judgemental support

Compassionate and non-judgemental support from staff and peers was the most consistently mentioned component of effective treatment services. It was reported in all but three studies [76, 80, 86], across both harm reduction and abstinence-based settings. Participants talked about the need to feel cared for and treated as individuals, by staff who would listen and be open and honest. Feeling cared for included having staff who looked out for them, provided encouragement, helped them feel accepted, were consistently available, went out of their way to help and who ‘*put their heart into helping*’ ([84]; p. 7). Compassion was all important, as illustrated below:

*Just somebody loving you heals you. Just somebody taking interest in what you’re doing heals you. Just saying that person’s name, taking your time out for them, it makes a person – it fills the soul, it fills the heart. The people here mainly need compassion.* ([71]; p. 852)

*… you could just show a little more compassion and gentleness. Understand that good people are also addicts … Give them a chance to heal and get better* ([74]; p. 689)

Conversely, participants talked about their experiences of feeling mistreated by disrespectful and uncompassionate staff, and being perceived as ‘*nothing but a junkie*’ ([78]; p. 627), as an ‘alien’ ([67]; p. 202), or as ‘addicts’ and ‘criminals’. Some also reported racism:

*Sometimes, when you’re in a hospital … and you’re an Aboriginal person … you know there’s a lot of racism in the hospital … They mistreat you and they don’t care … I think if I was treated equally like the other patients were being treated, like human beings and not mistreated, I would [stay] … treat them for who they are and not just because we’re Aboriginal people and drug addicts.* ([74]; p. 690)

Compassion and non-judgemental support included peer, practical and emotional support in harm reduction and abstinence-based settings, as reported in 11 studies [33, 67–70, 73, 77, 82–86, 88]. Being in close proximity to those with similar circumstances brought people together, providing supportive relationships which were also perceived as helping to prevent relapse for those who were abstinent [70, 73, 77, 82, 83, 85, 86, 88]. Participants talked about feeling at ease with the people around them because they could understand their situations and experiences. Peer support provided inspiration, hope and opportunities to engage with those further along in their recovery journey [67, 82]. In two studies of Managed Alcohol Programmes [70, 73], participants (mostly male) talked about peers as ‘family’:

*Everybody seems to support each other … the staff and the clients, they treat you like family … We try to help each other*. ([70]; p. 121)

Being compassionate also included realising what people needed and providing it through practical support, including food and non-alcoholic drinks; access to clean clothes and medication, and opportunities for tending to personal hygiene; travel expenses; help with appointments and finding doctors; support with benefits and budgeting, and gaining housing [67, 70, 86]. Neale and Stevenson ( [68]; p. 83) reflected on the varied support needs of their participants, including access to college, employment and housing, and ‘ultimately becoming part of society again’.

Emotional support was also viewed as important by participants in seven studies [33, 67, 69, 70, 82, 84, 88] and included access to formal counselling and support to manage traumatic experiences [67, 82]. Informal emotional support included being able to talk about daily concerns and receiving guidance in a non-judgemental/empathetic manner [67, 70, 84], enabling people to become more positive. Such support was discussed as being required in harm reduction and abstinence-based settings [33, 67, 69, 70, 82, 84, 88].

(iii) Importance of time

Participants talked about treatment needing to be long enough in duration for them to avoid relapse/move into recovery [33, 72, 84, 86, 87]. In two studies [86, 87], participants (all women) reported the need for ongoing support after their abstinence-based treatment ended. Neale and Kennedy and Salem et al. [67, 86] both discussed the benefits of an aftercare programme as a way of ensuring a supportive network to prevent relapse. Lengthy or continuous support was often considered necessary and could be provided in the form of safe housing, such as in Housing First settings. Women in Baird et al.’s [87] study talked about feeling ill-equipped for life outside of a shelter, and were concerned that lack of support after 90 days of an intensive abstinence-based programme would result in relapse. Perreault et al.’s [72] study of a 3-year peer support harm reduction housing programme reported that participants considered this to be of insufficient length:

*… [the programme] ends after three years. After, I’m supposed to have studied or worked, but that’s not easy. I don’t know if in three years I’ll be capable of working and finding an inexpensive apartment … it worries me a lot, the ‘after’ here … It took me six months to sober up and another six to stabilise. I don’t count my first year as looking for work or even possibly returning to school, I count it as just coming down to earth … The longer it lasts, the happier I’ll be.* ([72]; p. 357)

(iv) Having choices

Enabling people to feel that they had a choice about their treatment was reported as beneficial in seven studies [33, 68, 70, 74, 75, 78, 81]. Participants wanted to be treated as individuals with particular needs and be able to set their own goals, rather than experience a ‘*one size fits all*’ approach ([63]; p. 334). They described past experiences where they did not feel that they had choices:

*They really cover a whole wide gamut … that really gives the individual a lot of options. These other programmes are so set in stone. It’s not even a maze, it’s just a straight line and you gotta follow it, where Help Centre just has some good things and so many different pathways you can take to achieve what you want to achieve for yourself… they want you to know that the focus is on the individual*. ([78]; p. 630)

The desire for individualised care means flexibility in service delivery. For example, some participants experienced periods of abstinence in a harm reduction setting (Managed Alcohol Programme) because they were able to choose to stop drinking on their own terms [70]. In another study, the different needs of participants receiving counselling was highlighted: some preferring group settings and others one-to-one [33].

(v) Opportunities to (re)learn how to live

Across 14 studies [67, 69–74, 78, 80–83, 85, 86], treatment was seen as providing opportunities for clients to learn skills to support them to live their lives away from problematic patterns of substance use, which would also help stabilise their lives, including their housing. The majority of these studies were harm reduction-oriented, but there was also a sense of the need for these opportunities in abstinence-based settings. (Re)learning life-skills included using a computer, developing a hobby, cooking or participating in meaningful activities such as art, gardening, group trips and other classes. This provided structure and purpose to the day and enabled participants to build their personal identities, alleviate boredom and distract them from thinking about drugs/alcohol [67, 83, 86]:

*The programme is … teaching us to be in a home. You know, not like what we’re used to, out on the street. Like re-learning how to be in a house with responsibilities: got to make your bed, do your laundry, sweep, wash the floor, do dishes, and of course, we’re starting to cook. Most of us I think are just re-learning domestic things that you would normally do in a home. It’s another one of the benefits that we get living here*. ([73]; p. 7)

This point echoes findings reported in relation to practical support (see section above) on helping people to re-learn life skills. Evans et al.’s [70] participants talked about the challenges they experienced in learning how to live in a residential, harm reduction setting relating to understanding roles and routines. In another study, men and women highlighted the challenges of learning to live in a Housing First accommodation, highlighting the pressure, either real or perceived, of engaging with harm reduction approaches, as a result of previous negative experiences in other settings where substance use was penalised:

*The first year I would crack a beer in my own house and look around for the cops. And, I thought the whole year there was going to be a snag, and I was going to get kicked out for sure*. ([71]; p. 849)

There was general recognition that having goals and hope for the future was beneficial. Working on a range of goals was also important [67]. Addressing homelessness and substance use were perceived as essential first steps, but the value of smaller goals was also highlighted [78]. People reported wanting more responsibility for their lives, including seeking employment, reflecting that sometimes they were: ‘*still treated like kids … They don’t give us a chance to do it, so we’ll leave here without having that experience*’ ([72]; p. 358), although other participants reported having had different experiences: ‘*They turned my life around by showing me I’m my own person and helping me realise for once in my life I have choices and decisions*’ ([82]; p. 18).

Developing these life skills appeared to require participants to achieve some stability in their lives. There was a sense that effective treatment, both in terms of harm reduction and abstinence-based approaches, helped with this stability through providing structure, routine, autonomy and meaning in life:

*I am doing my daily routine quite well, making sure I get up in the morning and don’t just stay up watching shit TV until like four o‘clock in the morning. So I think I’m better now, better equipped to get up and do something during the day, like a normal human being*. ([69]; p. 85).

Whilst reciprocally translating findings, a refutational translation gradually emerged relating to the desire for stability. This translation was noted in first-order participant data in 11 studies of harm reduction and abstinence-based approaches [33, 69, 71–74, 78, 81, 83, 85, 86], but was only specifically noted in second-order author interpretations in five studies [68, 72, 73, 78, 83]. Thus, the level of importance attached to the desire for stability by study participants and authors markedly differed: authors often over-looked this when reporting and discussing their most significant findings, despite its centrality for service users.

### Line-of-argument synthesis

**Fig. 2:**
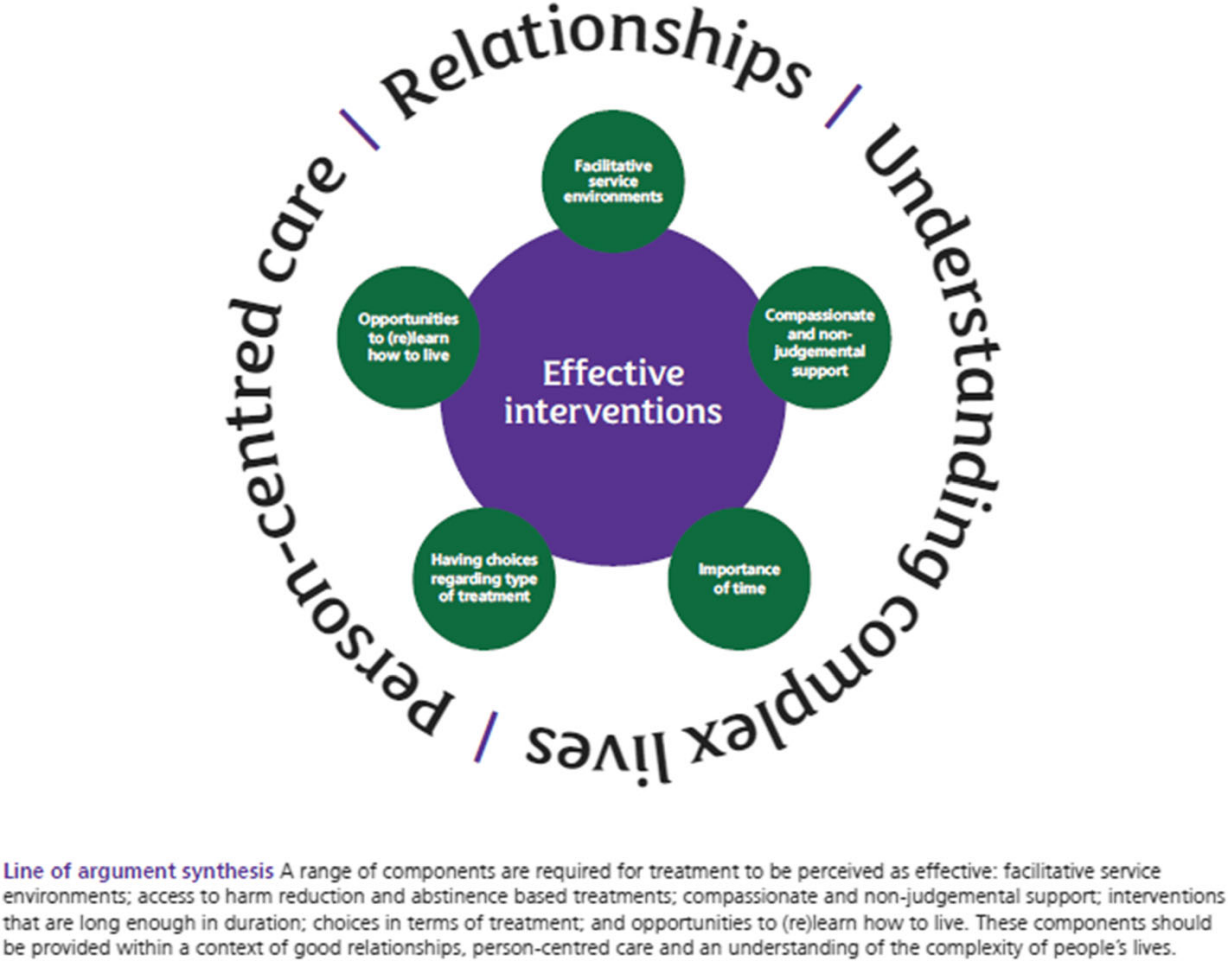
Components of effective substance use treatment from the service user perspective

From translation of findings across the 21 studies, a new line-of-argument emerged enabling creation of a model illustrating our new understanding of the components of effective treatment from the service user perspective (Fig. 2).

For treatment to be perceived as effective by those experiencing homelessness, several essential components are required: facilitative service environments; compassionate and non-judgemental support, including, if possible, the provision of support by people with lived experience; interventions that are long enough in duration, and offer continuity of support; having choices regarding treatment type (such as harm reduction and abstinence-based interventions); and opportunities to (re)learn how to live. Most importantly, these components should be provided within a service context which enables good relationships, person-centred care and an understanding of the complexity of people’s lives.

Service and treatment environments should be facilitative, staffed by people who are non-judgemental, compassionate, respectful and well-trained. It was apparent in some studies that provision of support by staff with previous lived experience of homelessness and problematic substance use was particularly beneficial to service users and thus has much potential. Services must ensure that they do not increase people’s risks of harm as a result of environments that do not take into account people’s experiences of trauma [89]. Support should be long enough in duration for people to gain stability, to avoid relapse and to move into (self-defined) recovery. Practical, peer and emotional support should be delivered without stigma, where trust, mutual respect and collaboration is fostered between those delivering and using services. It is important to note that, whilst some of these components may appear obvious or even taken for granted, these are not necessarily present or prioritised in current service delivery as we note above. While these key components are not radical concepts in and of themselves, they would, we believe, if implemented and financed consistently, provide a radically different experience for those using services.

Across many of the studies [33, 70, 71, 73, 74, 79, 83], male and female participants appeared to prefer services aligned with a harm reduction philosophy. For many, the culture of harm reduction is providing people with positive experiences of effective treatment. However, there were elements of both harm reduction and abstinence-based interventions that were perceived as effective. The intervention that people engage with will depend on individual circumstances, so access to a range of interventions with opportunities to choose which approach suits them best is required, which is a key principle of harm reduction [36, 40].

There should also be opportunities for people to (re)learn life skills, and partake in activities such as cooking, shopping, budgeting and to access education and employment, to help them reintegrate and grow in confidence, and these should be provided in conjunction with suitable, safe and secure housing. These opportunities should be identified on an individual basis: some may need parenting skills or knowledge on how to build and sustain relationships; others may need skills on sustaining housing. This requires goal setting and realistic timescales. While not discussed by participants in the included studies, occupational engagement can also improve outcomes for those experiencing homelessness [90].

For many, engaging with treatment will be challenging, so service providers must understand the complexity of people’s lives, and how their circumstances will affect engagement. Providing these key components within the context of person-centred care is essential. A facilitative environment, enabling people to develop trust and relationships with staff, should enable engagement with treatment and activities.

## Discussion

We synthesised the findings of 21 qualitative studies, reported in 23 papers that explored components of problematic substance use treatment deemed to be effective by adults experiencing homelessness in three countries. Our findings provide fresh insight into what is considered effective and why, from this viewpoint. We have highlighted several key components of effective treatment, as well as highlighting the importance of stability for participants, which has often been overlooked by authors. Of interest, those authors who noted the need for stability were writing about harm reduction approaches, suggesting stability may be perceived differently when considering harm reduction and abstinence-based treatment. This novel finding highlights the benefit of synthesising individual qualitative studies in a ME, identifying a component of treatment that is likely to be important to those experiencing homelessness, but possibly overlooked by those working in and researching the field. Our line-of-argument and model emphasise the key components which enable people to engage effectively with treatment and move on with their lives in positive and less harmful ways, including into (self-defined) recovery.

Harm reduction and abstinence-based treatments featured in the reviewed studies and all were reported by participants as having some degree of effectiveness. In several studies [33, 70, 71, 73, 74, 79, 83], participants preferred harm reduction-orientated services where they had opportunities to set individualised goals, rather than having to achieve and maintain abstinence. Often, harm reduction and abstinence-based treatments are understood as being located at different ends of a continuum, something that can be reinforced by service providers, policy and strategic decision makers and academic researchers. The findings of this review highlight that people who are homeless who also require services for problematic substance use want and need a range of interventions that belie this apparent separation. Harm reduction services provide a crucial way of engaging those who find high-threshold services inaccessible and meet people ‘where they are at’ [36], yet abstinence-based treatments should also be made available when people are open and ready to stop active use (either for the short or longer term). Harm reduction is pragmatic, compassionate and incremental, which are elements discussed by participants in the studies synthesised as essential to effective treatment. Our view is that these often opposing approaches should be understood as inherently complementary [91].

Individuals are likely to have different needs and desires relating to their substance use at different time points, so a continuum of services and approaches should be therefore be made available and offered without judgement. Those people experiencing homelessness should not feel that the only way to access a safe space to stop drinking or using drugs is in settings in which abstinence is expected or enforced. Instead, substance use services/treatment settings need to support people to stop using substances safely, as required, without any assumption of continued abstinence. As described by Rhodes [92], there are particular environments that increase people’s risk of drug harms, including a lack of suitable housing, high threshold services and access to appropriate treatment, as well as stigmatisation and drug laws. Thus, services must not increase people’s risk of harm. Thus, a truly flexible system which provides both harm reduction and abstinence-based approaches is recommended for people experiencing homelessness.

Importantly, people need choice regarding which intervention/treatment is best for them, and to have access to a range of approaches offered over a period lasting long enough to help them to achieve stability and prevent relapse. In our review, time was important, with interventions being considered effective when they went beyond formal treatment, either in terms of extended treatment approaches or in the provision of safe housing, such as Housing First. It is also likely that people’s choices may change over time, depending on their circumstances. This can also mean that their choices of harm reduction or abstinence-based approaches can also change over time, depending on which approach is deemed most appropriate to their current needs.

One aspect of effective treatment that came through strongly was the need to offer people opportunities to develop skills and hobbies that can help them to live a life off the streets. Acquisition of life skills arguably requires a certain degree of stability to be maintained. While such activities are most likely to be part of residential programmes, they also need to be embedded into community-based interventions. Online interventions can also provide opportunities to develop these skills. This finding fits with the concept of ‘recovery capital’ [45], where recovery from problematic substance use includes developing meaningful activities and creating additional sources of social support [93, 94], and aligns with studies exploring the role that client participation in life skills counselling/support can have on alcohol-specific and general approach coping [95].

This review identified that *how* an intervention is delivered is critically important. For those experiencing homelessness and problematic substance use, engagement with all forms of treatment or service can be particularly problematic due to judgemental attitudes of others and stigma [21, 43]. Regardless of the service approach, staff must be non-judgemental, and supportive relationships should be core. As Horvath et al. [96] highlight in their meta-analytic review, the alliance-outcome relationship is one of the strongest and most robust documentable predictors of treatment success making it one of the most important influences on individual psychotherapy outcomes. Alliance quality can reflect the collaborative dimension of therapy, as well as the importance of practitioners responding non-defensively to client behaviours. Better outcomes can be expected when the worker or therapist affirms and conveys unconditional warmth and liking for their client [97–99]. Meier and colleagues [100] reported similar findings in their review, with therapeutic alliance being a consistent predictor of engagement and retention in drug treatment.

### Implications from the review for policy, practice and research

Treatment for problematic substance use can be relatively short, with a minimum of 3 months being recommended [94], although longer-term treatment and continued support is considered beneficial [101]. In our review, participants considered an effective intervention to be one which provided long-term treatment, and ongoing support, to help them to achieve stability. The need for longer-term treatment duration is consistent with findings from other studies in which extended treatment is associated with improved outcomes [102, 103]. Ongoing support through aftercare tends to be provided following initial, more intensive, treatment, and includes mutual aid support groups as well as formal case management, home visits, and therapeutic contacts via telephone/face-to-face check-ups, in individual or group settings. According to Lash and colleagues [104], increasing the duration of care to at least a year, providing monitoring, actively reaching out to engage clients in ongoing care and using incentives can be beneficial.

Our findings therefore connect with broader evidence suggesting the need for development and evaluation of longer-term treatment and aftercare models, to avoid relapse, enhance stability, and enhance the likelihood of a range of positive outcomes, within both harm reduction and abstinence-based interventions. It is important to note that in our review, the requirement for lengthy treatment and aftercare came across strongly in the words of participants but was far less discernible within original author interpretations. Thus, the importance of stability may be underreported in the literature. For those experiencing homelessness and problematic substance use, the need for longer-term interventions and aftercare support is not surprising, given the myriad of challenges they faced. While the included studies did not specify a desired length of treatment, there was recognition that the longer support continues, the better. This desire from participants is in conflict with the reality of services globally, where austerity and systematic underfunding and cuts to services put pressure on services to discharge people as quickly as possible. This appears to be particularly problematic in the UK [25–27], but can also be seen in other countries. Thus, more research is required to identify the optimal length of treatment duration for those experiencing homelessness and problematic substance use.

Many of the factors identified by participants as essential to an effective intervention resonate with the concept of Psychologically Informed Environments (PIEs; 97), a psychological framework designed to ensure services respond to the needs of those experiencing homelessness. The traumatic experiences that people using services have had, and the ensuing emotional impact, lie at the core of this approach, with people’s coping strategies, including problematic substance use, being understood in this context [105, 106]. The physical environment and staff training are two key components of PIEs [89] and were highlighted by participants in 11 studies in our review. Service providers can make improvements to their environment in order to support those who find it hard to engage with mainstream health or substance use services, through the use of flexible drop-ins, improved kitchen and dining facilities, notice boards, lighting and décor [89].

Substance use treatment for those experiencing homelessness is a complex issue and therefore requires a complex, flexible, inter-agency response. For some, standalone interventions may facilitate engagement with treatment but are unlikely to enable individuals to maintain their recovery. For many, their housing situation complicates their ability to engage in treatment, so providing services that address their substance use along with other needs is vital. Interventions such as Managed Alcohol Programmes, transitional housing and Housing First provide individuals with a home to live in as well as access to a range of health and support services.

It is important to note that these findings are based on a particular group of people accessing treatment for problematic substance use when they are experiencing homelessness. There are many missing voices from the studies we reviewed, including those who identify as lesbian, gay, bisexual, transgender and other sexual identities (LGBT+); those not engaged in services; and participants from a wider range of countries. Such groups are likely to experience additional problems accessing and engaging with effective treatment [107–109]. Other voices, whilst present, were hard to perceive, especially women and those from ethnic minority communities. We therefore caution that the components identified in this review may not be relevant to the wider population of people with homelessness and problematic substance use concerns. More research is needed to identify components of effective treatment from those who were not included in the synthesised studies.

### Strengths and limitations

Details of our strengths and limitations are shown in Additional file 1. This novel ME has highlighted several components of effective treatment that are likely to have been neglected using other review methods, particularly the finding regarding the need for stability. Throughout the review, many steps were taken to enhance rigour: all stages of study searching, screening, quality appraisal, data extraction and analysis were checked for accuracy by at least two people, with regular team meetings held to reflect on the process and outcomes. Additionally, this ME is among the first to use the eMERGe reporting guidance [61] aimed at enhancing review quality and transparency. The reviewers consisted of four White Scottish/British women with backgrounds in social science/psychology, mental health/substance use and adult nursing; three work within the field of substance use. All four reviewers had significant experience in conducting qualitative research in a range of topics. ‘Sense-checking’ our findings with people with lived experience is not usual practice in ME; however, this step gave additional opportunities for critical reflection on our findings, line-of-argument and model by providing feedback from three ‘experts by experience’ (all White Scottish/British, two men and one woman). While we did not include people with lived experience throughout the review, we did involve stakeholders with relevant experience at various stages, including project planning. The findings resonated with their experiences.

It is important to note that our findings and model are based on the views of participants in included studies, which represent a relatively narrow view of those likely to be experiencing homelessness and problematic substance use. This paper deliberately focused on the experiences of adults over 18 years of age, and so excludes the experiences of younger people. Most of the studies (*n* = 18) were conducted in the USA and Canada, which may limit the transferability of the findings to other settings, particularly given differences in terms of the support provided to and treatment of those experiencing homelessness in terms of housing, healthcare (including substance use treatment), criminal justice system and welfare payments [110]. In conducting the ME, we specifically paid attention to participants’ characteristics in studies, such as gender and ethnicity as well as the country in which the study was conducted, in order to establish whether different groups reported different experiences or refuted our translations. However, a lack of diversity in the studies and in the geography of the research meant that opportunities to identify disconfirming cases were limited.

Five papers were excluded from review, either due to a lack of sufficient first-order data for translation or because the study did not fully meet the inclusion criteria [41, 111–114]. Inclusion of these studies is unlikely to have altered our findings and model; for example, these studies report similarities in terms of access to support [41, 113, 114]; the importance of having choices [41, 111]; feelings of safety and stability [41, 112]; and the different experiences of harm reduction and abstinence-based services [112, 114]. Study participants are similar to those in our included studies: mostly male; no sexual identity reported; and, of the studies reporting ethnicity, participants were mostly Black [111] or Indigenous Canadians [112]. In the North American context, the issue of culturally competent and respectful care for Indigenous people is particularly important [43]. This issue is not discussed in detail in the present review, however, because it did not emerge as a strong theme within our analysis (because it did not feature in the studies we were synthesising). Additionally, eight studies focused on alcohol, seven on alcohol and drugs and three on drugs. Finally, as with all qualitative research, throughout this review we have expected and addressed researcher bias; from the studies, and from our own reflections and processes.

## Conclusion

This novel ME extends the current evidence base by providing an understanding of the components required for effective problematic substance use treatment from the perspective of those experiencing homelessness. In line with implementation science, the components identified may promote the application of interventions in real-world settings. In particular, it seems that *how* treatment is provided is more important than the particular interventions people receive. Ensuring that people receive treatment in a facilitative environment, with staff who are non-judgemental, compassionate and respectful is critical. Opportunities to develop skills and to (re)learn how to live a life away from homelessness and substances further support effective treatment. In view of the importance of the desire for stability voiced by study participants, treatment and care should be provided for as long as is required by an individual, with continuing support or aftercare post-treatment. Our view is that these core components collectively represent an urgent call for a radical reorientation of services towards meeting the needs of individuals with multiple needs. Further research is needed to understand the views of a wider range of individuals, including those from minority groups or who are currently not engaged in services, for whom both experiences and views on what is needed may be quite different.

## Supplementary information

**Additional file 1.** Details of our methods as informed by the eMERGe meta-ethnography reporting guidance.

**Additional file 2.** Quality appraisal.

**Additional file 3.** Details of excluded studies.

**Additional file 4.** Example concept map.

## Data Availability

The datasets used and/or analysed during the current study are available from the corresponding author on reasonable request.
