# Supplementary material for: What constitutes effective problematic substance use treatment from the perspective of people who are homeless? A systematic review and meta-ethnography
Source: Harm Reduct J. 2020 Jan 31;17:10. doi: 10.1186/s12954-020-0356-9 (PMC6995160; doi:10.1186/s12954-020-0356-9)
Supplement: Supplementary file 1 — Additional file 1. Details of our methods as informed by the eMERGe meta-ethnography reporting guidance. [file 12954_2020_356_MOESM1_ESM.docx]

**Additional File 1. Details of our methods as informed by the eMERGe meta-ethnography reporting guidance**

| This review drew on Noblit and Hare’s (48) seven phases of meta-ethnography: (1) getting started; (2) deciding what is relevant to the initial interest; (3) reading the studies; (4) determining how the studies are related; (5) translating the studies into one another; (6) synthesising translations; and (7) expressing the synthesis. |
| --- |
| **Phase 1: Getting started**  There are multiple methods of Qualitative Evidence Synthesis (QES) and Meta-ethnography (ME) is just one approach. We specifically chose to use ME as the most appropriate QES approach for this study because it was designed to bring together purely qualitative research. It takes into account the research contexts in primary studies and goes beyond thematic analysis of the original studies to produce new interpretations, models or theories. ME was therefore best suited to our study as our intention was to develop a new model or theory to explain effective treatment for problematic substance use for those experiencing homelessness.  The review team included those with expertise in substance use and related service provision (HC, TP and JM are part of the Salvation Army Centre for Addiction Services and Research at University of Stirling, funded to conduct research and knowledge exchange activities in the field of problematic substance use). NR was experienced in ME and was a member of the team which developed the eMERGe ME reporting guidance.  Prior to developing our review protocol, initial discussion with stakeholders in the field identified our broad ME topic and helped us define key terms (see below) for use within the review.   - ‘Homelessness’ was defined as a lack of suitable, stable and permanent housing, including those who are sleeping rough, living in hostels, with family/friends or in residential treatment programmes. Those ‘at risk of homelessness’ may include people who are likely to lose their own home, due to substance use, poverty and other factors. - ‘Effectiveness’ is often used to refer to quantitative outcomes but we used this term to reflect interventions and services perceived by participants as beneficial to them or as improving their outcomes in any way. This term reflected the language used by our diverse stakeholders e.g. they talked about effectiveness of treatment rather than acceptability. It also recognises the importance of ‘re-contextualising’ effectiveness in the context of QES (42), to reflect interventions that are useful to those receiving them. - ‘Treatment’ was defined as meaning a diverse range of interventions, ranging from harm reduction to abstinence-based approaches. This definition of treatment is based on the policy and practice context in which the review was conducted (Scotland). It also reflects our own experiences of working in services for people who are homeless and within drug and alcohol services, as well as being involved in studies where harm reduction is understood to be a vital form of treatment and is viewed on a continuum from abstinence-based treatments. - ‘Adults’ are defined as those aged over 18 years. Studies involving younger age groups were excluded as the focus of the review was on adults.   Preliminary searching identified that relevant qualitative research studies were available for synthesis. Preliminary searching also identified that we should focus our review on studies reporting the voices of those experiencing treatment from the perspective of those receiving rather than delivering such services; a knowledge gap the ME was well suited to address. We also believed that focusing on the views of treatment users would enable in-depth and original insight to be produced and would facilitate development of a conceptual model from the perspective of service users which could then be used to inform practice and policy. Stakeholder discussion confirmed this narrower focus was a priority topic.  PROSPERO was checked to ensure no similar reviews were in progress. On developing our review protocol, including specifying our ME aims and review question (see methods sections), this was registered on PROSPERO (CRD42017069745). |
| **Phase 2: Deciding what is relevant**  Details of literature searching and screening: see methods section of paper. Details of search outcomes: see Figure 1.  Our search strategy was informed by SPIDER (55). Searching was comprehensive with electronic database searching for published literature (see Table 1 for search terms) and searching of websites (see Table 2) of key national/international organisations for grey literature. Grey literature searches were limited to research and evaluation reports published from 2007. Grey literature searching was limited to a 12 year period from when the review started to ensure that the most current sources were included in the study and because grey literature searching can be very time consuming. The published literature search covered a longer period because of the lengthier time for academic articles to be published and because it was feasible to search more years within the electronic databases.  Prior to conducting our search, we discussed our proposed search terms with a university librarian who considered this approach to be acceptable for our purposes. We chose to take a broad and inclusive approach to our search strategy, focusing on sensitivity rather than specificity. We felt that this was necessary because there is, for example, no standard definition of homelessness and we wanted to maximise our capture of relevant papers.  Literature searching was conducted by JM and HC in May 2019. JM searched all the databases (CINAHL, Criminal Justice Abstracts, Health Source, MEDLINE, PsycINFO, SocINDEX, Scopus and Web of Science). EMBASE was not searched as we had no access to this. Initial title and abstract screening of all potential items for inclusion was conducted by HC and JM who worked independently and then collaboratively to compare their screening outcomes. Any disagreements or uncertainty regarding screening was referred to the full team for discussion until consensus was reached. Possible items for inclusion in the ME had their full texts read (HC and JM) and 38 items were identified as meeting our initial criteria. Full text reading identified that seven of these papers focused on users of youth services which included those under 18 years of age as well older participants. Three papers also included adults with substance use and other mental health problems (dual diagnosis). After team discussion we decided it was not appropriate to include these papers in this ME but to retain them for separate synthesis and reporting to ensure the voices of these groups were not lost. Our initial study inclusion/exclusion criteria were therefore refined to indicate that these groups were excluded (see Box 1) and our PRISMA (Figure 1) amended accordingly. The reference lists of included papers were reviewed for other potential papers, but no further items were identified from these reference list citations.  On completion of Phase 2, 28 studies were identified for Phase 3. |
| **Phase 3: Reading included studies**  These 28 studies were (re-)read in full by all team members and quality appraised. Quality appraisal (using Critical Appraisal Skills Programme (CASP) checklist (57) was conducted (HC) as a means of systematically understanding the included papers in-depth. CASP assessment outcomes were discussed within the project team, as recommended by (42). See Additional File 2 for details of quality appraisal.  Quality appraisal and data extraction enabled very close reading and scrutiny of these papers and highlighted that some were not suitable for inclusion in Phases 4-6. Three papers (33,104,105) did not have sufficiently rich first order data (fewer than five quotes). In two other papers (103,106) some but not all participants appeared to meet our inclusion criteria (homeless/problematic substance use). Project timescales and the volume of data involved in the ME process were such that we were unable to contact these authors for further information. A team decision was made to exclude these five papers from translation (Figure 1 was updated to reflect this) but to retain them separately so that they could be reflected upon in Phase 7 to determine whether their inclusion would have altered our line-of-argument (LOA) synthesis.  Data extraction: characteristics of included studies were extracted as papers were quality appraised (led by HC, checked by JM) – see Table 3. First order (participant data) and second order data (author interpretation) from the 23 papers were extracted in preparation for Phases 4-6. First and second order data were extracted verbatim into separate word documents (HC) from across the full paper. This was done separately for each paper, in chronological order of year of publication. First and second order data word documents were then imported into NVivo and organised into two folders reflecting the different levels of data.  On completion of Phase 3, 23 papers from 21 studies were identified for inclusion in Phases 4-7. These were: 22 published papers (24,59,68–77,60,78,79,61–67), and one ‘grey’ literature study (80) (see Table 3 for characteristics of the included studies). Four papers were from two studies (65,68,69,77), meaning the findings from 21 studies were synthesised. |
| **Phase 4: Determining how studies related**  The 23 papers were initially compared by their characteristics (e.g. participants, methods and settings) to determine how they related – see Findings and Table 3.  These 23 papers were then related by their findings. Extracted data were line-by-line coded in NVivo (HC and JM) to identify themes and concepts as reported by original participants and original authors. Codes were then entered into Excel matrices, with our coded categories along the top and study details on the side. As coding progressed, refinements were made to the matrices. The matrices enabled us to determine how studies related by identifying similarities and differences between studies in their design (e.g. participants, setting) and findings as we could establish which studies had reported first and/or second order data on each category. Matrices also allowed identification of relationships between coding structures and/or hierarchies. Coding, matrix development, referral to full texts for data contextual information and reflective team discussion enabled determination of how studies related according to their original participant and author findings (all authors but led by HC). For example, by identifying which studies reported findings on service user support and which did not. |
| **Phase 5: Translating studies into one another**  Translation flowed seamlessly from Phase 4 and involved constant comparison between studies and reflective discussion within the team.  The 23 included papers were first reciprocally translated using the review questions as a priori categories – what is perceived as effective problematic substance use treatment from the perspective of those who are homeless, and how does treatment work. Creating a tree of nodes and a concept map showing each data category from Phase 4 enabled us to see how the studies answered the review questions (see Additional File 4 for an example concept map). This approach to reciprocal translation worked well and although challenging it was relatively straightforward to translate studies according to whether they provided insight into what interventions worked, or did not, from the service user perspective. The process of reciprocal translation also enabled refutational translation because as we became deeper immersed in the data, further differences between and within studies were gradually revealed. When differences were noted, we reflected on possible explanations for these, such as study setting or participant gender, and we constantly returned to the data or study full texts to help understand these differences and disconfirming cases. For example, we observed that the desire for stability featured more often in participant data than it did in author interpretations (see Findings). |
| **Phase 6: Synthesising translations**  Synthesising translations was very complex and time consuming, involving frequent team meetings to critically reflect on our emerging synthesis of what was being reported (or not) across the data. Gradually we moved past looking at findings from individual studies and the a priori categories to enable our new third order interpretation to develop that went beyond describing the themes in the original studies and initial translations. For example, translations relating to staff attributes and treatment philosophy were gradually synthesised into ‘facilitative service environments’.  During synthesis careful consideration was given to the levels of interpretation in the studies, examining participant interpretations (quotes), the author interpretations (data analysis, reported findings and conclusions/ recommendations), and the interpretations developed by us when considering the papers as a whole. To ensure our interpretation was based on the data and awareness of context, synthesis was an iterative process and we constantly re-referred to the concept maps, patterns in the matrices, and full texts, throughout this process. We were also careful to ensure our synthesis reflected our nuanced understanding of the translated data, for example, ensuring that participant desire for stability which had been under-reported by original authors was adequately reflected within our interpretation.  Our new line-of-argument (LOA) developed slowly to reflect our translations and was refined following critical discussion within the team over a number of months. Gradually we created a model visually representing the narrative of our LOA. |
| **Phase 7: Expressing the synthesis**  *Findings*: these are presented as narrative, a model, supporting tables, and additional files.  *Limitations*: See also Discussion section of paper. This was a small study conducted by staff (HC/TP/JM) employed in a research centre funded by The Salvation Army. No additional funding was received for this study. The small team (four reviewers) were all white Scottish females with backgrounds in social science, mental health/substance use and adult nursing. ME is an interpretative process, and the research team member backgrounds informed development of their final LOA and model. Another research team that included individuals from different disciplines, countries and of different gender and ethnicity would have brought different perspectives to this interpretive process. It is possible their final LOA and model may have differed from ours. However, across the ME phases, we were rigorous in ensuring that our interpretation was based on the data in the included studies.  The review team did not consist of those with lived experience of homelessness and substance use but the team was supported by a small group of people with lived experience with whom we shared the initial findings and model (two in an informal group meeting with HC and one via email; see Strengths section of Discussion). Our decision to involve people with lived experience in Phase 7 was innovative (this is not required in ME) as their views and feedback facilitated reflection on our findings, the included studies and the LOA. The voices of those with lived experience were also vital in helping us contextualise our findings.  Included studies were conducted in the UK (n=3), USA (n=11), and Canada (n=7). Women were particularly under-represented amongst participants, with only three studies specifically targeting them. Additionally, the included studies did not fully describe other protected characteristics of participants. It is therefore not known to what extent minority populations are represented amongst these study samples. It is also important to note that all participants were recruited from services and none directly from the street so these findings reflect the views of those who were already engaged, to some extent, with services. We only included data from the perspectives of those experiencing homelessness, rather than from staff providing those services, because synthesising data from both groups was out of the scope of this review. Project timescales were such that we did not have time to contact authors of studies meeting our inclusion criteria to request additional data.  *Strengths*: TP has direct experience of working with those who have experienced homelessness and substance use. HC led on the research activities but the team met regularly to review processes and outcomes (for example, to critically reflect on alternative explanations for our translations and LOA synthesis). NR has specialist expertise in ME including membership of the eMERGe project which developed ME reporting guidance to enhance quality and transparency of reports (53). All authors have considerable experience of conducting qualitative research and conducting reviews. The development of our review was influenced by those working in the field of problematic substance use and homelessness, with stakeholders being involved in the development of the review question and in contextualising the findings, including specialist groups in the field and those with lived experience.  Steps were taken to enhance the quality of this review: for example, following development of our LOA synthesis we returned to the literature to consider whether any studies published after our searches were completed met our inclusion criteria and, if so, whether their findings refuted ours and could have resulted in a different LOA synthesis if included in our review. We also returned to the five studies that met our inclusion criteria that were excluded in Phase 3. Again, these studies reported similar findings, of the need for choices when in treatment (33,103,105); the importance of the service environment (33,104); the need to (re)learn skills (103,106); and the need for support, both in the long- and short-term (33,103-106). There were issues raised in these studies that were not captured in our review, for example, difficulties in accessing methadone (33); challenges around hospital stays (103); and the need for age-specific services (106), which reflect the particular context of these studies.  *Implications for practice:* See more detailed section in the paper. Our findings enabled us to develop a new conceptual model and provide novel insight into the particular components of that model that those experiencing homelessness find helpful when accessing treatment for problematic substance use. In particular, it is ‘how’ treatment is provided that appears to be more important than the particular interventions that people receive. A service that is welcoming, with staff who are compassionate, well trained and non-judgemental; having time, choices and support; the opportunity to develop more stability in life; and the opportunities to learn or relearn skills, were appreciated by participants. Services can ensure that their environment is as welcoming and non-judgemental as possible, and that people are treated with respect and as worthy human beings. A range of support can be provided to support people’s needs. Treatment should be provided for as long as is required, with follow-up support post formal treatment. People should be treated as individuals and provided with choices to set their own goals. Finally, services should provide people with opportunities to develop skills and engage in meaningful activities. |
| **References as per manuscript** |
