# Supplementary material for: What constitutes effective problematic substance use treatment from the perspective of people who are homeless? A systematic review and meta-ethnography
Source: Harm Reduct J. 2020 Jan 31;17:10. doi: 10.1186/s12954-020-0356-9 (PMC6995160; doi:10.1186/s12954-020-0356-9)
Supplement: Supplementary file 2 — Additional file 2. Quality appraisal. [file 12954_2020_356_MOESM2_ESM.docx]

**Additional File 2. Quality appraisal**

| **Source** | **Statement of aims** | **Appropriate method** | **Appropriate design** | **Appropriate recruitment** | **Appropriate data collection** | **Relationship between researcher and participant considered** | **Ethical issues considered** | **Rigorous data analysis** | **Statement of findings** | **How valuable is the research?** | **Total** |
| --- | --- | --- | --- | --- | --- | --- | --- | --- | --- | --- | --- |
| Neale & Kennedy (2002) | Yes | Yes | Yes | Yes | Yes | Not clear | Not clear | Yes | Yes | Yes | 8/10 |
| Lee & Petersen (2009) | Yes | Yes | Yes | Yes | Yes | Not clear | Not clear | Not clear | Yes | Yes | 7/10 |
| Rayburn & Wright (2009) | Yes | Yes | Yes | Yes | Yes | Not clear | Not clear | Yes | Not clear | Yes | 6/10 |
| Rayburn & Wright (2010) | Yes | Yes | Yes | Not clear | Yes | Not clear | Not clear | Not clear | Yes | Yes | 6/10 |
| Burkey et al. (2011) | Yes | Yes | Yes | No | Yes | Not clear | Yes | Yes | Yes | Yes | 8/10 |
| Kidd et al.  (2011) | Yes | Yes | Yes | Yes | Yes | Yes | Yes | Yes | Yes | Yes | 10/10 |
| Sznajder-Murray & Slesnick (2011) | Yes | Yes | Yes | Yes | Yes | Not clear | Yes | Yes | Yes | Yes | 9/10 |
| Collins et al. (2012a) | Yes | Yes | Yes | Not clear | Yes | Not clear | Not clear | Yes | Yes | Yes | 9/10 |
| Collins et al. (2012b) | Yes | Yes | Yes | Not clear | Yes | Not clear | Not clear | Yes | Yes | Yes | 7/10 |
| Thickett & Bayley (2013) | Yes | Yes | Yes | Yes | Yes | Yes | Not clear | Yes | Yes | Yes | 9/10 |
| Baird et al.  (2014) | Yes | Yes | Yes | Yes | Yes | Not clear | Not clear | Not clear | Yes | Yes | 7/10 |
| Neale & Stevenson (2014a) | Yes | Yes | Yes | Yes | Yes | Not clear | Not clear | Yes | Yes | Yes | 8/10 |
| Neale & Stevenson (2014b) | Yes | Yes | Yes | Yes | Yes | Not clear | Not clear | Yes | Yes | Yes | 8/10 |
| Salem et al. (2013) | Yes | Yes | Yes | Yes | Yes | Not clear | Not clear | Yes | Yes | Yes | 8/10 |
| Evans et al. (2015) | Yes | Yes | Yes | Yes | Yes | Not clear | Not clear | Not clear | Yes | Yes | 7/10 |
| Clifasefi et al. (2016) | Yes | Yes | Yes | Yes | Yes | Not clear | Not clear | Yes | Yes | Yes | 8/10 |
| Collins et al. (2016) | Yes | Yes | Yes | Yes | Yes | Not clear | Yes | Yes | Yes | Yes | 9/10 |
| McNeil et al. (2016) | Yes | Yes | Yes | Yes | Yes | Not clear | Yes | Yes | Yes | Yes | 9/10 |
| Pauly et al. (2016) | Yes | Yes | Yes | Yes | Yes | Not clear | Yes | Yes | Yes | Yes | 9/10 |
| Perreault et al. (2016) | Yes | Yes | Yes | Yes | Yes | Not clear | Yes | Yes | Yes | Yes | 9/10 |
| Chatterjee et al. (2018) | Yes | Yes | Yes | Yes | Yes | Not clear | Not clear | Yes | Yes | Yes | 8/10 |
| Crabtree et al. (2018) | Yes | Yes | Yes | Yes | Yes | Not clear | Yes | Yes | Yes | Yes | 9/10 |
| Pauly et al. (2018) | Yes | Yes | Yes | Yes | Yes | Not clear | Not clear | Yes | Yes | Yes | 8/10 |
