# Supplementary material for: What constitutes effective problematic substance use treatment from the perspective of people who are homeless? A systematic review and meta-ethnography
Source: Harm Reduct J. 2020 Jan 31;17:10. doi: 10.1186/s12954-020-0356-9 (PMC6995160; doi:10.1186/s12954-020-0356-9)
Supplement: Supplementary file 3 — Additional file 3. Details of excluded studies. [file 12954_2020_356_MOESM3_ESM.docx]

**Additional File 3. Details of excluded studies**

| **Paper** | **Reason for exclusion** | **Excluded at phase** |
| --- | --- | --- |
| Bozinoff N, Small W, Long C, DeBeck K, Fast D. Still “at risk”: An examination of how street-involved young people understand, experience, and engage with “harm reduction” in Vancouver’s inner city. Int J Drug Policy. 2017;45:33–9. doi: 10.1016/j.drugpo.2017.05.006 | Participants were young people/youth | Phase 2 |
| Buccieri K. Harm reduction as practice: Perspectives from a community of street youth and social service providers. Soc Dev Issues. 2010;32(3):1–15. | Participants were young people/youth | Phase 2 |
| Christiani A, Hudson AL, Nyamathi A, Mutere M, Sweat J. Attitudes of homeless and drug-using youth regarding barriers and facilitators in delivery of quality and culturally sensitive health care. J Child Adolesc Psychiatr Nurs. 2008;21(3):154–63. doi: 10.1111/j.1744-6171.2008.00139.x. | Participants were young people/youth | Phase 2 |
| Garrett S, Higa D, Phares M, Peterson P, Wells E, Baer J. Homeless youths’ perceptions of services and transitions to stable housing. Eval Program Plann. 2008;31(4):436–44. doi: 10.1016/j.evalprogplan.2008.04.012. | Participants were young people/youth | Phase 2 |
| Hudson A, Nyamathi A, Slagle A, Greengold B, Griffin D, Khalilifard F, et al. The power of the drug, nature of support, and their impact on homeless youth. J Addict Dis. 2009;28(4):356–65. doi:10.1080/10550880903183026. | Participants were young people/youth | Phase 2 |
| Kozloff N, Cheung AH, Ross LE, Winer H, Ierfino D, Bullock H, et al. Factors influencing service use among homeless youths with co-occurring disorders. Psychiatr Serv. 2013;64(9):925–8. doi: 10.1176/appi.ps.201200257. | Participants were young people/youth with dual diagnosis | Phase 2 |
| Mancini MA, Wyrick-Waugh W. Consumer and practitioner perceptions of the harm reduction approach in a community mental health setting. Community Ment Health J. 2013;49(1):14–24.  doi: 10.1007/s10597-011-9451-4. | Focus of paper is on dual diagnosis | Phase 2 |
| Nyamathi A, Hudson A, Mutere M, Christiani A, Sweat J, Nyamathi K, et al. Drug use and barriers to and facilitators of drug treatment for homeless youth. Patient Prefer Adherence. 2007;1:1–8. | Participants were young people/youth | Phase 2 |
| Padgett DK, Henwood B, Abrams C, Davis A. Engagement and retention in services among formerly homeless adults with co-occurring mental illness and substance abuse: Voices from the margins. Psychiatr Rehabil J. 2008;31(3):226–33. doi:10.2975/31.3.2008.226.233 | Focus of paper is on dual diagnosis | Phase 2 |
| Padgett DK, Henwood BF. Qualitative Research for and in Practice: Findings from Studies with Homeless Adults Who Have Serious Mental Illness and Co-Occurring Substance Abuse. Clin Soc Work J. 2012;40(2):187–93. | Focus of paper is on dual diagnosis | Phase 2 |
| Cornes M, Manthorpe J, Joly L, O’Halloran S. Reconciling recovery, personalisation and Housing First: Integrating practice and outcome in the field of multiple exclusion homelessness. Heal Soc Care Community. 2014;22(2):134–43; doi:10.1111/hsc.12067. | Lack of first order data (participant quotes) | Phase 3 |
| Matheson C, Liddell D, Hamilton E, Wallace J. Older people with drug problems in Scotland: A mixed methods study exploring health and social support needs. 2017. <http://www.sdf.org.uk/wp-content/uploads/2017/06/OPDP-mixed-methods-research-report-PDF.pdf> Accessed 1 Aug 2019 | Focus was not specifically on homelessness; not all participants had experienced homelessness. | Phase 3 |
| Neale J. Homelessness amongst drug users: A double jeopardy explored. Int J Drug Policy. 2001;12(4):353–69; doi:10.1016/S0955-3959(01)00097-4. | Lack of first order data (participant quotes) | Phase 3 |
| Raven MC, Carrier ER, Lee J, Billings JC, Marr M, Gourevitch MN. Substance use treatment barriers for patients with frequent hospital admissions. J Subst Abuse Treat. 2010;38(1):22–30; doi:10.1016/j.jsat.2009.05.009. | Study did not specifically focus on those who are using substances and it is difficult to distinguish between whether findings relate to substance use, mental health or other factors | Phase 3 |
| Vallance K, Stockwell T, Pauly B, Chow C, Gray E, Krysowaty B, et al. Do managed alcohol programs change patterns of alcohol consumption and reduce related harm? A pilot study. Harm Reduct J. 2016;13(1):1–11; doi:10.1186/s12954-016-0103-4. | Lack of first order data (participant quotes) | Phase 3 |
