# Supplementary figures and images for: What constitutes effective problematic substance use treatment from the perspective of people who are homeless? A systematic review and meta-ethnography
Source: Harm Reduct J. 2020 Jan 31;17:10. doi: 10.1186/s12954-020-0356-9 (PMC6995160; doi:10.1186/s12954-020-0356-9)

**Additional File 4. Example concept map**


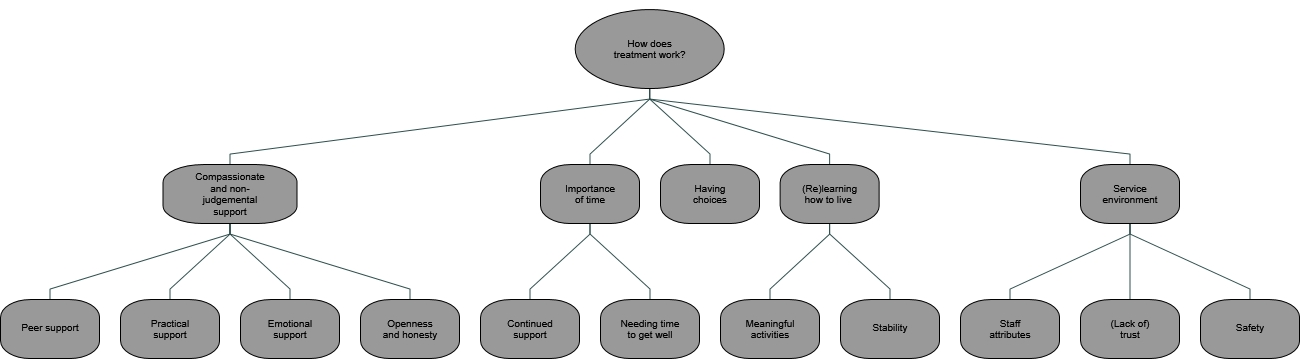

Supplement: Supplementary file 4 — Additional file 4. Example concept map. [file 12954_2020_356_MOESM4_ESM.docx]
